# High Environmental Ozone Levels Lead to Enhanced Allergenicity of Birch Pollen

**DOI:** 10.1371/journal.pone.0080147

**Published:** 2013-11-20

**Authors:** Isabelle Beck, Susanne Jochner, Stefanie Gilles, Mareike McIntyre, Jeroen T. M. Buters, Carsten Schmidt-Weber, Heidrun Behrendt, Johannes Ring, Annette Menzel, Claudia Traidl-Hoffmann

**Affiliations:** 1 ZAUM – Center of Allergy & Environment, Member of the German Center for Lung Research (DZL), Technische Universität München/Helmholtz Center, Munich, Germany; 2 Department of Ecology and Ecosystem Management, Ecoclimatology, Technische Universität München, Freising, Germany; 3 Department of Dermatology and Allergy, Technische Universität München, Munich, Germany; 4 Christine-Kühne-Center for Allergy Research and Education (CK Care), Davos, Switzerland; Cincinnati Children's Hospital Medical Center, University of Cincinnati College of Medicine, United States of America

## Abstract

**Background:**

Evidence is compelling for a positive correlation between climate change, urbanisation and prevalence of allergic sensitisation and diseases. The reason for this association is not clear to date. Some data point to a pro-allergenic effect of anthropogenic factors on susceptible individuals.

**Objectives:**

To evaluate the impact of urbanisation and climate change on pollen allergenicity.

**Methods:**

Catkins were sampled from birch trees from different sites across the greater area of Munich, pollen were isolated and an urbanisation index, NO_2_ and ozone exposure were determined. To estimate pollen allergenicity, allergen content and pollen-associated lipid mediators were measured in aqueous pollen extracts. Immune stimulatory and modulatory capacity of pollen was assessed by neutrophil migration assays and the potential of pollen to inhibit dendritic cell interleukin-12 response. *In vivo* allergenicity was assessed by skin prick tests.

**Results:**

The study revealed ozone as a prominent environmental factor influencing the allergenicity of birch pollen. Enhanced allergenicity, as assessed in skin prick tests, was mirrored by enhanced allergen content. Beyond that, ozone induced changes in lipid composition and chemotactic and immune modulatory potential of the pollen. Higher ozone-exposed pollen was characterised by less immune modulatory but higher immune stimulatory potential.

**Conclusion:**

It is likely that future climate change along with increasing urbanisation will lead to rising ozone concentrations in the next decades. Our study indicates that ozone is a crucial factor leading to clinically relevant enhanced allergenicity of birch pollen. Thus, with increasing temperatures and increasing ozone levels, also symptoms of pollen allergic patients may increase further.

## Introduction

Epidemiological studies show an increasing trend in allergies, leading to a major health problem. Reasons discussed for this trend include a westernized life style with diminished immune stimulation [1] and anthropogenic air pollution [2], [3]. Particularly, irritant gases and diesel exhaust particles have been shown to exert adjuvant or aggravating effects on sensitisation and elicitation phases of allergic immune responses [4], [5]. As underlying mechanisms, effects on cells of the immune system as well as epithelial barrier disruption are discussed [6]. However, pollutants in ambient air do not only impact humans but also the allergen-carrier itself, i.e. the plant and its pollen. Therefore, the question arises whether the observed increase in allergic diseases in the western world might in part be explained by modified allergenicity of pollen caused by urbanisation and paralleled climate change. These environmental changes − higher temperature, in combination with higher concentrations of specific anthropogenic pollutants − lead to higher tropospheric ozone concentrations. In this scenario UV-radiation delivers the energy for ozone generation, but besides this, higher temperatures can also lead to an increase in ozone formation promoted by emission of highly reactive hydrocarbons from vegetation and evaporation processes [7]. Climate extremes are often observed in cities, which function as heat islands and can be regarded as a mirror of future climate [8]. However, urbanisation is not only characterised by higher temperatures, but also by higher levels of pollutants like particulate matter, carbon dioxide (CO_2_) or nitrogen dioxide (NO_2_). In this respect, it has to be considered that ozone does not show the same distribution as other pollutants [7], [9]. Ozone is a secondary pollutant whose formation underlies complex interactions, depending on the presence of precursors, degrading substances, temperature and UV-radiation. The main precursors are nitrogen oxides (NOx) and volatile organic compounds (VOCs). Especially NOx can be found at high concentrations in urban areas. NO, in turn, rapidly degrades the ozone generated in urban areas, especially during night-time. In rural areas, in contrast, we observe an accumulation of ozone due to lower NO levels and higher biogenic emissions [7]. Some studies already addressed the question of how pollutants affect the allergen carrier, showing that single pollutants strongly differ in their effects [10], [11]. The current study expands these observations by analyzing one of the most relevant allergen producers – the birch tree – in its natural environment. Hereby, we work with real exposure conditions to analyse relevant factors under natural circumstances. We take into account the parallel occurrence of different environmental factors as well as mechanisms of plant adaptation. Recent studies showed that pollen do not only release allergens, but also non-allergenic compounds such as pollen-associated lipid mediators (PALMs) (reviewed in [12]), which have been shown to exert immune modulatory and stimulatory effects. Allergenicity, thus, was evaluated in a holistic approach also taking adjuvant factors into account.

This study aimed at understanding how long-term increases in urbanisation and concomitant increases in pollution might influence pollen allergenicity, and how this might translate into immune cell activation and symptoms of pollen-allergic patients.

## Materials and Methods

### Ethic Statement

The ethical committee of the Technical University of Munich approved the study, and volunteers were enrolled after written informed consent. The study was carried out on private land and the owners of the land gave permission to conduct the study on these sites.

### Sampling of Birch Pollen

Catkins were collected from birch trees located in the greater area of Munich (n = 40; see Fig. S1). Sampling of catkins took place during the birch flowering season in spring 2010. Their developmental stages were assessed by an adopted and extended code of the BBCH (Biologische **B**undesanstalt, **B**undessortenamt und **Ch**emische Industrie) [13]. The code included 12 different developmental stages, starting with winter rest (BBCH 50) and ending with end of flowering (BBCH 69). The stages of collection were BBCH 60 (single catkins sporadically emit pollen) and BBCH 61 (10% of the catkins emit pollen). After collection, catkins were air-dried, counted and weighed and pollen was extracted by sieving.

### Preparation of Aqueous Pollen Extracts for Bet v 1, LTB_4_ and PGE_2_ ELISA

Aqueous pollen extracts (APEs) were prepared in 0.1 M NH_4_HCO_3_, pH 8.1, as previously described [14]. For skin prick tests, APEs were prepared as described in [15]. APE concentrations given refer to the extraction of a given amount of pollen per mL of buffer before centrifugation (e. g. 1 mg pollen was extracted in 1 mL buffer), but do not refer to actual protein concentrations in the extracts.

### Bet v 1 ELISA

Bet v 1 levels were determined by sandwich ELISA as described in Buters et al. [14]. Bet v 1-specific antibodies MAK 2E10G6G7 and 4B10D10F8 were kindly provided by Joachim Ganzer, Allergopharma, Hamburg, Germany.

### PGE_2_ and LTB_4_ ELISA

Concentrations of the eicosanoid-like PALMs in APEs were measured by commercially available enzyme immunoassays for prostaglandin E_2_ and leukotriene B_4_ (GE Healthcare, Germany) according to the supplier’s protocol.

### Passive Sampling Method for Ozone and Nitrogen Dioxide (NO_2_) Determination

Passive samplers for ozone were provided and analysed by PASSAM AG, Männedorf, Switzerland. Passive sampling for ozone and NO_2_ were done in parallel. Passive sampling was carried out during the one-week period from May 11^th^ to 18^th^ 2010, i.e. 2 weeks after the last catkins were sampled. Measurements for all 40 birch trees were done in the same time frame and directly at the tree. It is supposed that the traffic volume over the period of 1 week is a representative mean for this location. This data was used to characterize the tree for a relative ozone exposure.

The nitrogen dioxide concentration was measured at the 40 sites according to Palmes’ principle [16]. Briefly, stainless steel meshes were immersed in a triethanolamine-aceton mixture and were air-dried for 10 minutes. Three coated meshes were brought into an air-tight tube. NO_2_ binds to the coated meshes by forming a triethanolamine-NO_2_-complex. NO_2_ adsorption was determined photometrically after one week of exposure.

### Calculation of Urbanisation Index

An urbanisation index (UI) based on CORINE Land Cover 2000 data (European Environment Agency 2000) was calculated using ArcGIS 9.3. This index reflects the proportion of predefined built up areas (e.g. continuous and discontinuous urban fabric, industrial or commercial units) within a radius of 2 km and thus can vary between 0 and 1; i.e. from a low (UI = 0) to a high (UI = 1) degree of urbanisation [17].

### Temperature Measurements

15 birch trees were provided with data recording devices measuring air temperature (HOBO U23-001, Onset Computer Corporation, Southern MA, USA). The devices were fixed in a radiation shield at the northern side of the trees in 3 m height. Air temperatures were recorded every 10 minutes, and daily temperature means were calculated. Temperature data were acquired between 1^st^ July 2009 and 5^th^ May 2010. The mean temperature for this period for each location was calculated.

### Blood Donors

Healthy, non-atopic blood donors without a history of allergic diseases were tested by RAST for sensitisation against common allergens including birch allergens. All subjects were tested negative and total IgE was <100 IU/ml. Volunteers did not take any medication for at least 15 days before blood sampling.

### Isolation and Culture of Monocyte-derived Dendritic Cells

Monocyte-derived dendritic cells (moDCs) were cultured from human peripheral blood monocytes as described by Gilles et al. [18]. Immature DCs were harvested on day 5 followed by stimulation with LPS (100 ng/ml) plus APEs from the highest (pollen^O3high^: mean ozone = 85 µg/m^3^, n = 2) and lowest (pollen^O3high^: mean ozone = 54 µg/m^3^, n = 2) ozone-exposed birch trees included in the study (mean ozone: 54 µg/m^3^ versus 85 µg/m^3^). After 24 h, supernatants were collected, and IL-12p70 release was measured by ELISA (BD Pharmingen, Heidelberg, Germany). The IL-12 response to APEs alone was measured exemplary in four APEs and virtually no IL-12 was detected (unstimulated: 18.7 pg/ml; APEs: median (min.-max.): 15.4 (9.9–20.8) pg/ml, n = 4) (data not shown). Viability of the cells after 24 h of culture was tested by propidium iodide staining and subsequent FACS analysis (see Fig. S4, supplementary material). Viability was not decreased by any of the conditions.

### Neutrophil Migration Assays

The chemotactic activity of APEs from the highest (pollen^O3high^: mean ozone = 85 µg/m^3^) and lowest (pollen^O3high^: mean ozone = 54 µg/m^3^) ozone-exposed birch trees included in the study was evaluated by measuring neutrophil migration through a 5 µm pore polycarbonate membrane (ChemoTx Disposable Chemotaxis System, NeuroProbe). Neutrophils were isolated from peripheral blood as described by Traidl-Hoffmann et al. [19]. APEs were pipetted into the bottom chambers and neutrophils (1×10^6^ cells/ml) were added to the top of the membrane. After 1 h of incubation the cell suspension was removed and cells that had transmigrated into the lower chamber were recovered and counted with a FACSCalibur (Becton Dickinson, Heidelberg, Germany).

### Skin Prick Tests

Birch allergic patients (n = 5) with a specific IgE >0.35 kU/l were pricked on their forearms with APE (10 mg/mL) prepared of pollen samples from the highest (pollen^O3high^: mean ozone = 85 µg/m^3^) and lowest (pollen^O3high^: mean ozone = 54 µg/m^3^) ozone-exposed birch trees of the study. Each sample was pricked in replicates of four (proximally and distally on the same arm, right and left arm), and the mean wheal and flare sizes were calculated out of these four measurements after 15 min.

### Statistics

Unpaired t-test was used for Gaussian distributed samples to determine statistically significant differences between groups. For non-Gaussian populations, the non-parametric Mann-Whitney U test was applied. The correlation coefficients (r) and 95% confidence intervals were calculated using the Pearson’s approach for Gaussian distributions and the Spearman’s approach for non-parametric correlation. Additionally, we applied linear multiple regression analyses based on stepwise variable selection to test which environmental factor is most important for pollen allergenicity. To analyse cell assays, the area under the curve (AUC) was determined and the Wilcoxon matched-pairs signed-ranks test was applied. Prick tests were also analysed by the Wilcoxon matched-pairs signed-ranks test. P values of <0.05 were considered significant (*). **: p<0.01, ***: p<0.001. Statistics were done with Graph Pad Prism 5, San Diego, CA, USA.

## Results

### Correlation Analysis of Urbanisation Related Factors and Relationship to Pollen Allergenic Potential

**Figure 1:**
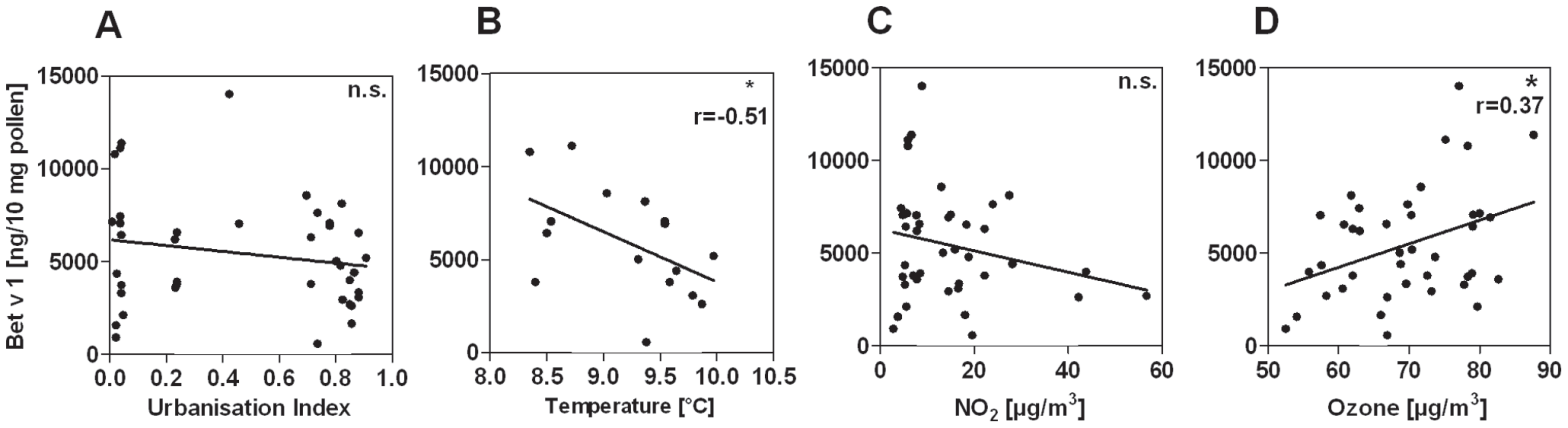
Bet v 1 content of birch pollen in trees against different, urbanisation-related environmental conditions. No significant correlation could be observed between Bet v 1 content and urbanisation index (A; n = 40) or NO_2_ concentration (B; n = 40**)**. Bet v 1 showed a significant and negative correlation with temperature (C; n = 16) and was positively correlated with site-specific ozone levels (D; n = 40). *: p<0.05.

No significant correlation was detected between Bet v 1 content of the pollen specimens and urbanisation index (UI) or NO_2_ concentration (Fig. 1A, C; n = 40). However, a negative correlation could be observed for Bet v 1 content and temperature (Fig. 1B; r = -0.51; p = 0.042; n = 16). In contrast, ozone was positively correlated with Bet v 1 content. (r = 0.37; p = 0.017; Fig. 1D; n = 40).

**Figure 2:**
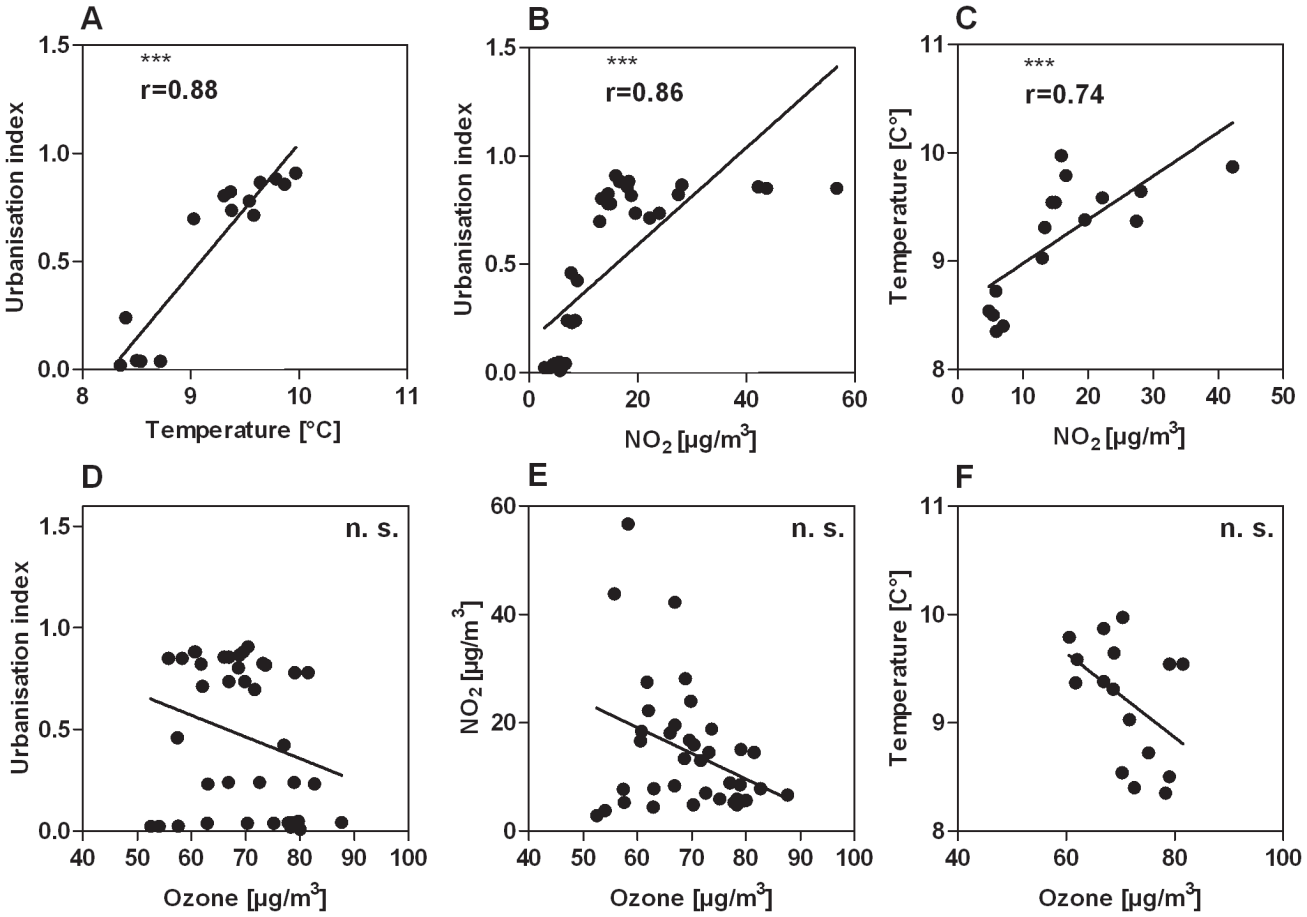
Scatter plots of different environmental parameters. A significant correlation was observed between the parameters urbanisation index (UI) and temperature (A; n = 16), (B) UI and NO_2_ concentration (n = 40) and (C) temperature and NO_2_ concentration (n = 16). Ozone was not correlated with either (D) UI (n = 40), (E) NO_2_ (n = 40) or (F) temperature (n = 16); ***: p<0.001.

Sites of birch trees were analyzed for environmental parameters. By correlation analyses the relationship of the determined parameters was investigated. A highly significant and positive correlation of the variables UI (n = 40), temperature (n = 16) and NO_2_-concentration (n = 40) could be observed (Fig. 2 A, B, C). In contrast, ozone (n = 40) was not statistically associated with any of these parameters (Fig. 2 D, E, F).

To investigate whether ozone or temperature is the factor influencing pollen Bet v 1 content, we applied multiple regression analyses based on stepwise variable selection. Indeed, the role of the independent variable ozone for predicting Bet v 1 content was superior, since temperature has been excluded in the linear model, yielding in an R^2^ of 27.6% (data not shown).

**Figure 3:**
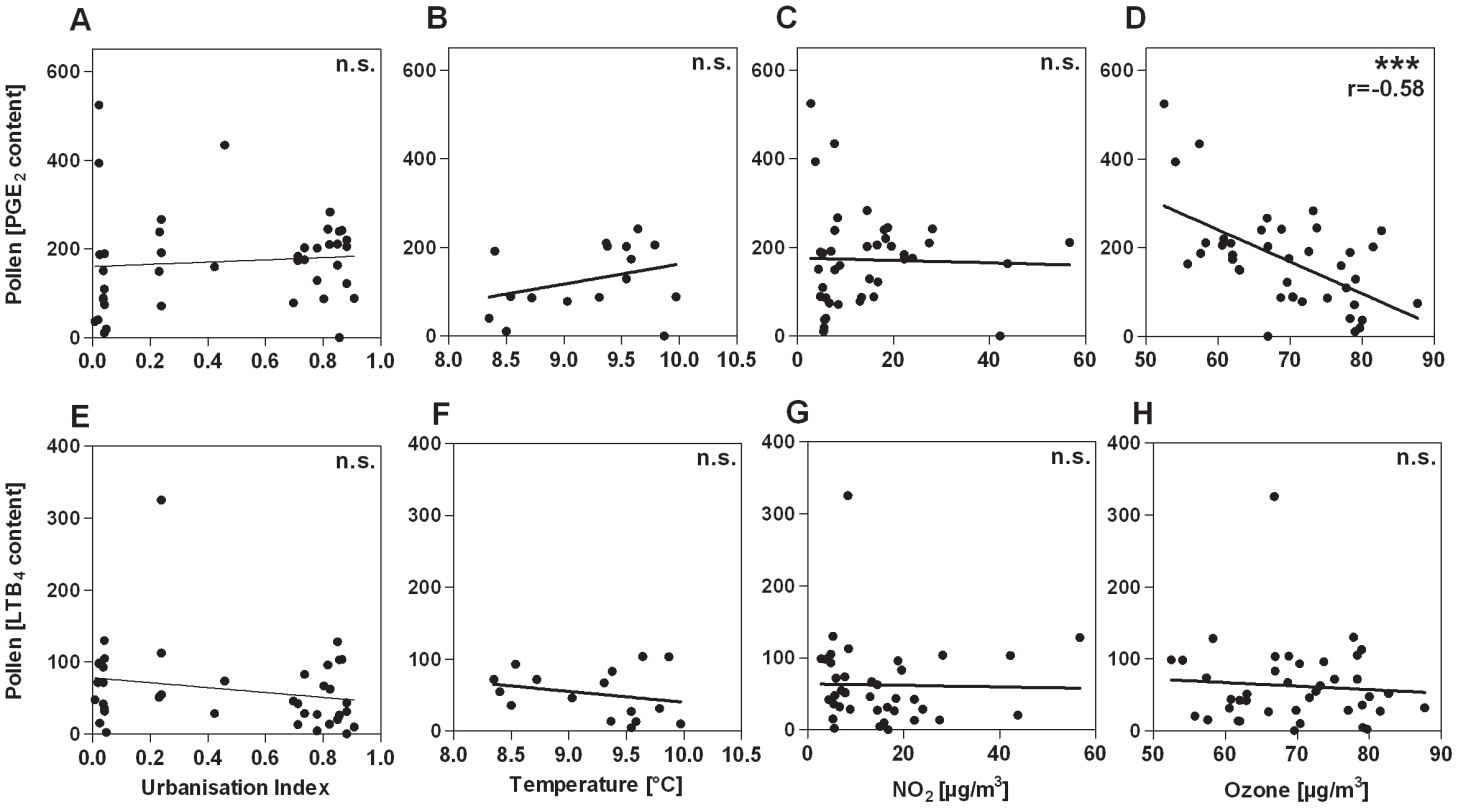
Content of PALMs in pollen samples plotted against different urbanisation-related environmental conditions. No correlation was seen between PALM_PGE2_ and UI (A; n = 40), temperature (B; n = 16) or NO_2_ concentration (C; n = 40). A significant association of high ozone concentrations and low PALM_PGE2_ contents was observed (D; n = 40). PALM_LTB4_ did not show any significant correlation. Neither UI (E; n = 40), nor temperature (F; n = 16), NO_2_- (G; n = 40) and ozone (H; n = 40) were related to the content of PALM_LTB4_. ***: p<0.001.

To determine immune stimulatory and modulatory potential of the birch pollen specimens, we analysed the content of PALMs in the different pollen extracts. Prostaglandin E_2_-like PALMs, termed PALM_PGE2_, harbour the immune modulatory PALMs [20], [21]. No statistically significant correlation was observed between PALM_PGE2_ contents and UI (n = 40), temperature (n = 16) or ambient NO_2_ (n = 40) (Fig. 3A, B, C). Instead, PALM_PGE2_ content was negatively correlated with ambient ozone (n = 40) levels (r = -0.58; p<0.0001; Fig. 3D). The LTB_4_-like PALMs harbour the chemotactic, immune stimulatory PALMs [19]. PALM_LTB4_ contents were not correlated with either UI (n = 40), temperature (n = 16), NO_2_ (n = 40) or ozone (n = 40) levels (Fig. 3E, F, G, H).

### Immune-stimulatory and Immune-regulatory Capacity of Pollen Samples

**Figure 4:**
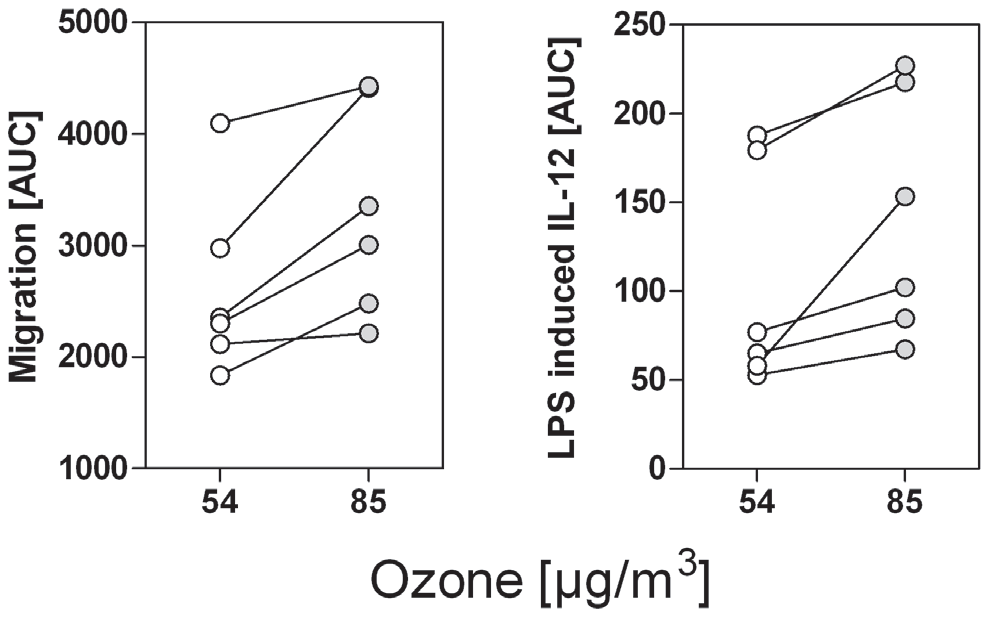
Immune stimulatory versus immune modulatory potential of pollen samples from higher- and lower-ozone-exposed birch trees. Aqueous extracts (APEs) of birch pollen sampled from high and low ozone exposed trees were chosen for neutrophil migration assays and stimulation of monocyte derived dendritic cells. APEs were applied in 3 concentrations and the AUC was calculated. Higher ozone-exposed pollen induced stronger neutrophil chemotaxis compared to pollen samples from lower ozone–exposed trees (**A**). In contrast, birch pollen from lower ozone-exposed trees were more potent in inhibiting the LPS-induced release of IL-12p70 from human monocyte-derived dendritic cells (**B**). APEs were prepared from birch pollen sampled from higher ozone-exposed trees (n = 2; mean ozone: 85 µg/m^3^) and from lower ozone-exposed trees (n = 2; mean ozone: 54 µg/m^3^). All APEs were tested in n = 3 patients. *: p<0.05 (Wilcoxon matched-pairs signed-ranks test).

To assess whether the altered lipid compositions related to higher and lower ozone concentrations go along with differences in immune stimulatory and –modulatory potential, pollen from differentially ozone-exposed birch trees (mean ozone: 85 µg/m^3^ (pollen^O3high^) versus 54 µg/m^3^ (pollen^O3low^)) were chosen and analysed for induction of neutrophil chemotaxis and modulation of dendritic cell (moDC) cytokine secretion. Migration to pollen^O3high^ and pollen^O3low^ was tested in 3 concentrations (Fig. S4) and the AUC was calculated. Pollen^O3high^ induced significantly stronger neutrophil chemotaxis than pollen^O3low^. Neutrophil migration towards pollen extracts (median AUC (min.-max.)) were 3181 (2214–4429) for pollen^O3high^ versus 2327 (1839–4095) for pollen^O3low^; p = 0.03 (Fig. 4A). To test the immune modulatory capacity of differentially ozone-exposed pollen, moDCs were stimulated with LPS in the presence and absence of APE, and IL-12 was measured in the supernatants (LPS induced IL-12 response: median (min.-max.): 14000 (8928–14500) pg/ml). APEs were applied in 3 concentrations (Fig. S3) and AUC was calculated. As shown in Fig. 4B, pollen^O3high^ were less potent inhibitors of the moDC’s IL-12 response than pollen^O3low^. IL-12 release of dendritic cells (% of LPS; median AUC (min-max)) after stimulation with APE was 127.7 (67.3–227.0) for pollen^O3high^ versus 71.0 (52.8–187.7) for pollen^O3low^; p = 0.02.

**Figure 5:**
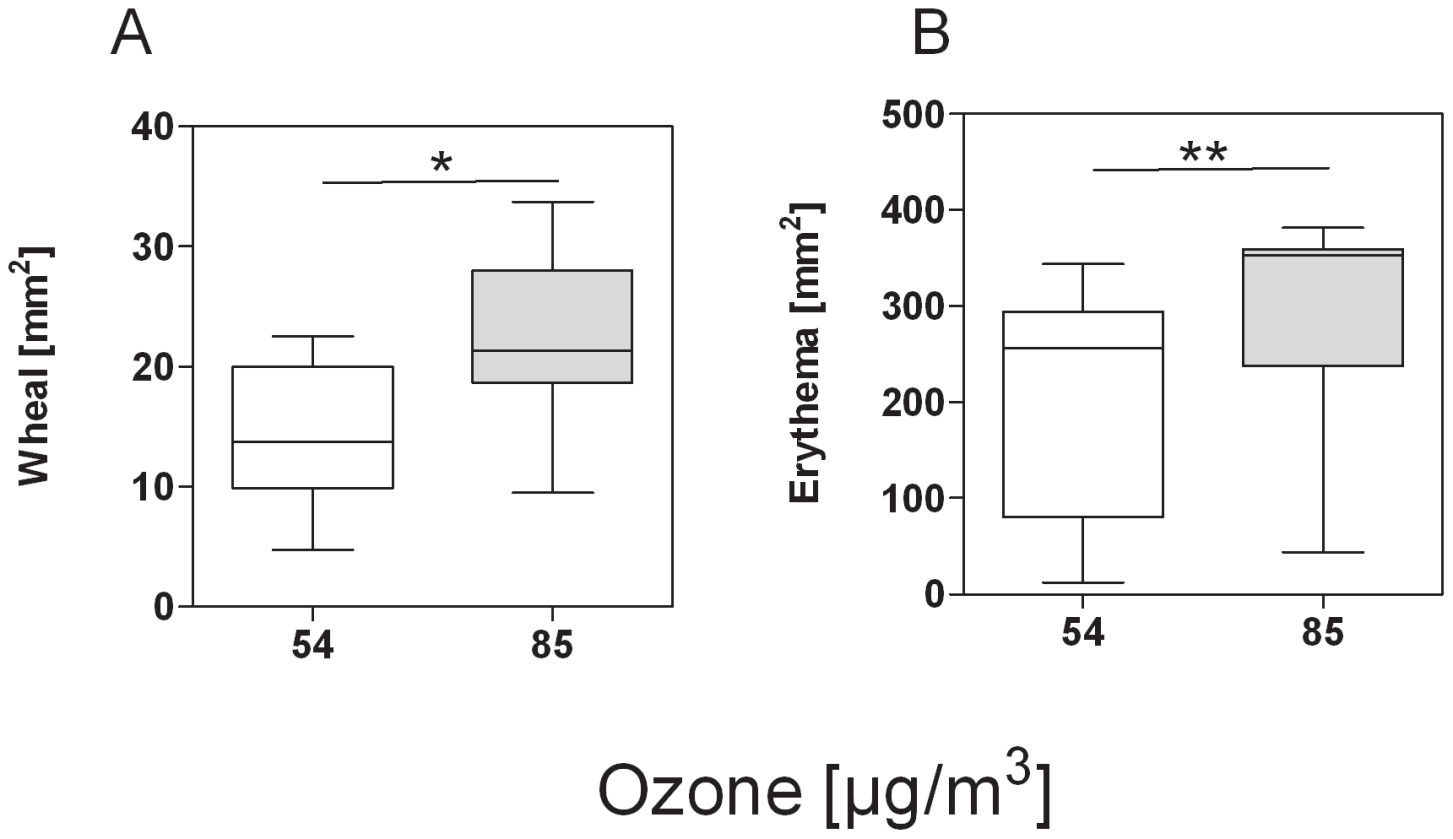
Cutaneous immune response towards pollen from higher and lower ozone-exposed birch trees. APEs were prepared from pollen sampled from higher ozone-exposed trees (n = 2; mean ozone: 85 µg/m^3^) and from lower ozone-exposed trees (n = 2; mean ozone: 54 µg/m^3^). All APEs were tested in n = 5 patients. Higher ozone-exposed pollen induced larger wheals (**A**) and flares (**B**) in skin prick tests compared to lower ozone-exposed pollen. *: p<0.05; **: p<0.01 (Wilcoxon matched-pairs signed-ranks test).

To test for clinical relevance of enhanced Bet v 1 levels, birch pollen allergic patients were subjected to skin prick tests with APEs prepared from differently exposed pollen. Wheal and flare sizes were significantly larger when patients were pricked with APEs prepared from pollen^O3high^. Wheal sizes (in mm^2^; median (min.-max.)) were 21.3 (9.5–33.8) for pollen^O3low^ and 13.8 (4.7–22.5) for pollen^O3high^; p = 0.02. Flare sizes (in mm^2^; median (min.-max.)) were 256.3 (12.4–343.8) for pollen^O3low^ and 353.1 (43.8–381.3) for pollen^O3high^; p = 0.005 (Fig. 5A, B). This is in line with higher Bet v 1 content of pollen^O3high^.

## Discussion

The present study reveals ozone exposure as important factor enhancing the allergenicity of birch pollen, with clinical relevance for susceptible individuals. Besides a positive relationship of the Bet v 1 content with ozone, we observed a negative association with temperature. Results of studies analysing the influence of temperature on Bet v 1 expression, however, are inconsistent. While Ahlholm et al. [22] reported a positive effect of high temperatures on Bet v 1 content, Helander et al. [23] indicated a negative association. The effect of temperature on the expression of Bet v 1 was also investigated by Tashpulatov et al. [24], using transgenic tobacco plants carrying a Bet v 1a-pomoter-reporter gene fusion. This study showed that warm temperatures positively regulate the activity of the Bet v 1 promoter. Our data indicate the opposite as we observed a negative correlation of temperature with Bet v 1. However, temperature and UI as well as NO_2_ concentration were highly and significantly related, while UI and NO_2_ did not correlate with Bet v 1. The number of entities for NO_2_ and UI were 40, while merely 16 data points for temperature were available. Considering the strong relationship of the parameters UI and NO_2_ with temperature it can be assumed that the correlation of temperature and Bet v 1 is observed by chance. Moreover when having a detailed look at the interrelationship of the environmental parameters of this study we also observed a non-significant negative association of ozone and temperature (Fig. 2F; p = 0.07; r = -0,46). This seems to be contradictory as ozone formation is associated with UV radiation and temperature [25]. However, ozone formation also depends on the presence of precursors (e.g. biogenic emissions) and degrading substances (e.g. NO). Rural areas are associated with lower temperatures. At the same time, the composition of ozone forming and -degrading factors in rural areas favor ozone accumulation. This might explain why we observe a negative correlation of ozone and temperature.

It might be hypothesized that a positive influence of ozone on Bet v 1 and the relationship of ozone and temperature implied a negative correlation of temperature and Bet v 1.

To confirm our suggestion, we applied multiple regression analyses based on stepwise variable selection. Indeed, the role of the independent variable ozone for predicting Bet v 1 content was superior, since temperature has been excluded in the linear model (data not shown).

Besides, Tashpulatov et al. [24] showed that abscisic acid, a stress- and development-related plant hormone, positively regulates the activity of the Bet v 1a promoter. This goes in line with our study showing that ozone, a major stress factor for plants, is associated with a higher Bet v 1 expression. Besides, also former studies in ragweed and grass species gave evidence that ozone impacts on the allergen transcript and content [26], [27]. In these studies, plants were exposed to defined ozone concentrations. In our study, trees were subjected to pollen sampling in their natural environment and under natural exposure conditions.

In a holistic approach analysing the allergenicity of birch pollen, we showed that elevated ozone exposure of birch trees was not only associated with increased allergen content in pollen but also with an altered composition of adjuvant PALMs. No relation of PALM_LTB4_ and PALM_PGE2_ content and the degree of urbanisation (UI), temperature or NO_2_ concentration was observed. This seems to be in contrast to a recent study [28]. However, this discrepancy is most likely explained by the fact that in the present study, pollen sampling was carried out at defined, distinct maturation stages. As demonstrated in Fig. S2, the content of PALMs in pollen grains differs profoundly depending on the maturation stage of pollen, as does the Bet v 1 content, confirming former results [29].

Notably, PALM_PGE2_ was significantly negatively associated to ozone concentrations. In our in vitro assays, pollen^O3high^ were significantly more chemotactic for neutrophils than extracts of pollen^O3low^. Since PALM_LTB4_ did not significantly differ in differently exposed pollen we hypothesize that other, up to now unknown, substances besides PALM_LTB4_ account for this effect. Moreover, Armstrong [30] could show that PGE_2_ is able to inhibit the chemotaxis of neutrophils in a concentration dependent manner. Consequently, lower concentrations of PALM_PGE2_ in pollen^O3high^ could also have contributed to enhanced neutrophil chemotaxis. In contrast, APEs prepared from pollen^O3low^ were significantly more efficient in inhibiting dendritic cell IL-12 secretion, in line with higher levels of immune modulatory PALM_PGE2_. Among the immune modulatory PALMs are plant isoprostanes identified as E_1_-phytoprostanes. E_1_-phytoprostanes inhibit the IL-12 response in maturing DCs [31], [32], finally licensing DCs to differentiate naïve CD4^+^ T cells into Th2 cells [32]. Transferring our data to allergy mechanisms, we hypothesize that higher PALM_PGE2_ concentrations in low-ozone areas might facilitate *de novo* sensitisation by providing Th2 promoting signals. Higher Bet v 1 concentration and less anti-inflammatory PALMs in pollen^O3high^ might in turn lead to pronounced allergic symptoms in already sensitized individuals.

In summary, urbanisation-related, anthropogenic environmental factors can influence birch trees to produce pollen with altered allergenic potential. Our study emphasizes the correlation of ozone exposure to the pollen content of allergen and non-allergenic, adjuvant factors. It is likely that future climate change with more frequent and intense warm spells [33] as well as increases in urbanisation and anthropogenic air pollutants such as NOx will further enhance the local accumulation of tropospheric ozone. As indicated by this study, ozone might – apart from direct adverse impacts on human health – lead to increased allergic symptoms *via* its impact on the allergen carrier.

## Supporting Information

Figure S1 **Locations of pollen sampling.** Birch pollen were sampled during the birch flowering season of 2010. Red dots represent urban trees, green dots rural trees. Background: CORINE Land Cover 2000 (EEA 2000), major classes: red = urban fabric, green = forest and pastures, yellow = arable land, blue = rivers, lakes (see www.eea.europa.eu/themes/landuse/interactive/clc-download for a complete legend).
(TIFF)

Figure S2 **Catkin maturation and allergenic potential of pollen. A:** Catkins of different maturation stages were collected at different time points from the same trees (BBCH-Code 51–52: n = 1; BBCH-Code 55–65: n = 5) and classified according to a BBCH-Code. Pollen were isolated from the catkins and aqueous pollen extracts were prepared. APEs were then analyzed for the presence of Bet v 1 and PALMs. The content of Bet v 1 peaked at maturation stages 60–61. Inversely to Bet v 1, levels of PALM_LTB4_ and PALM_PGE2_ were high in pollen from immature catkins and decreased during maturation. A concentration minimum of PALMs corresponded to a maximum in Bet v 1. BBCH-Code: 52: catkins increase in length and show green expansion cracks; 55: enhanced expansion cracks through further increase in length; 60: first catkins emit pollen (sporadically); 61: beginning of flowering: few catkins emit pollen; 65: full flowering: more than 50% of the catkins emit pollen; 67: flowering finishing: just a few catkins still emit pollen.
(TIFF)

Figure S3 **Immune stimulatory versus immune modulatory potential of high versus low ozone-exposed pollen samples.** Aqueous extracts (APEs) of birch pollen sampled from high and low ozone exposed trees were chosen for neutrophil migration assays and stimulation of monocyte derived dendritic cells. APEs were applied in 3 concentrations. Higher ozone-exposed pollen induced stronger neutrophil chemotaxis compared to pollen samples from lower ozone–exposed trees (**A**). In contrast, birch pollen from lower ozone-exposed trees were more potent in inhibiting the LPS-induced release of IL-12p70 from human monocyte-derived dendritic cells (**B**). APEs were prepared from birch pollen sampled from higher ozone-exposed trees (n = 2; mean ozone: 85 µg/m^3^) and from lower ozone-exposed trees (n = 2; mean ozone: 54 µg/m^3^). All APEs were tested in n = 3 patients. *: p<0.05 (Wilcoxon matched-pairs signed-ranks test).
(TIFF)

Figure S4 **Viability of moDCs after stimulation with LPS plus APEs from high- and low ozone-exposed pollen.** Viability of monocyte-derived dendritic cells (moDcs) after 24 h of stimulation with LPS (100 ng/ml) and APEs (1, 3, 10 mg/ml) was tested by propidium iodide staining and subsequent FACS analysis. APEs were prepared from birch pollen sampled from higher ozone-exposed trees (n = 2; mean ozone: 85 µg/m^3^) and from lower ozone-exposed trees (n = 2; mean ozone: 54 µg/m^3^). All APEs were tested in n = 3 patients.
(TIFF)
