# Supplementary figures and images for: High Environmental Ozone Levels Lead to Enhanced Allergenicity of Birch Pollen
Source: PLoS One. 2013 Nov 20;8(11):e80147. doi: 10.1371/journal.pone.0080147 (PMC3835901; doi:10.1371/journal.pone.0080147)

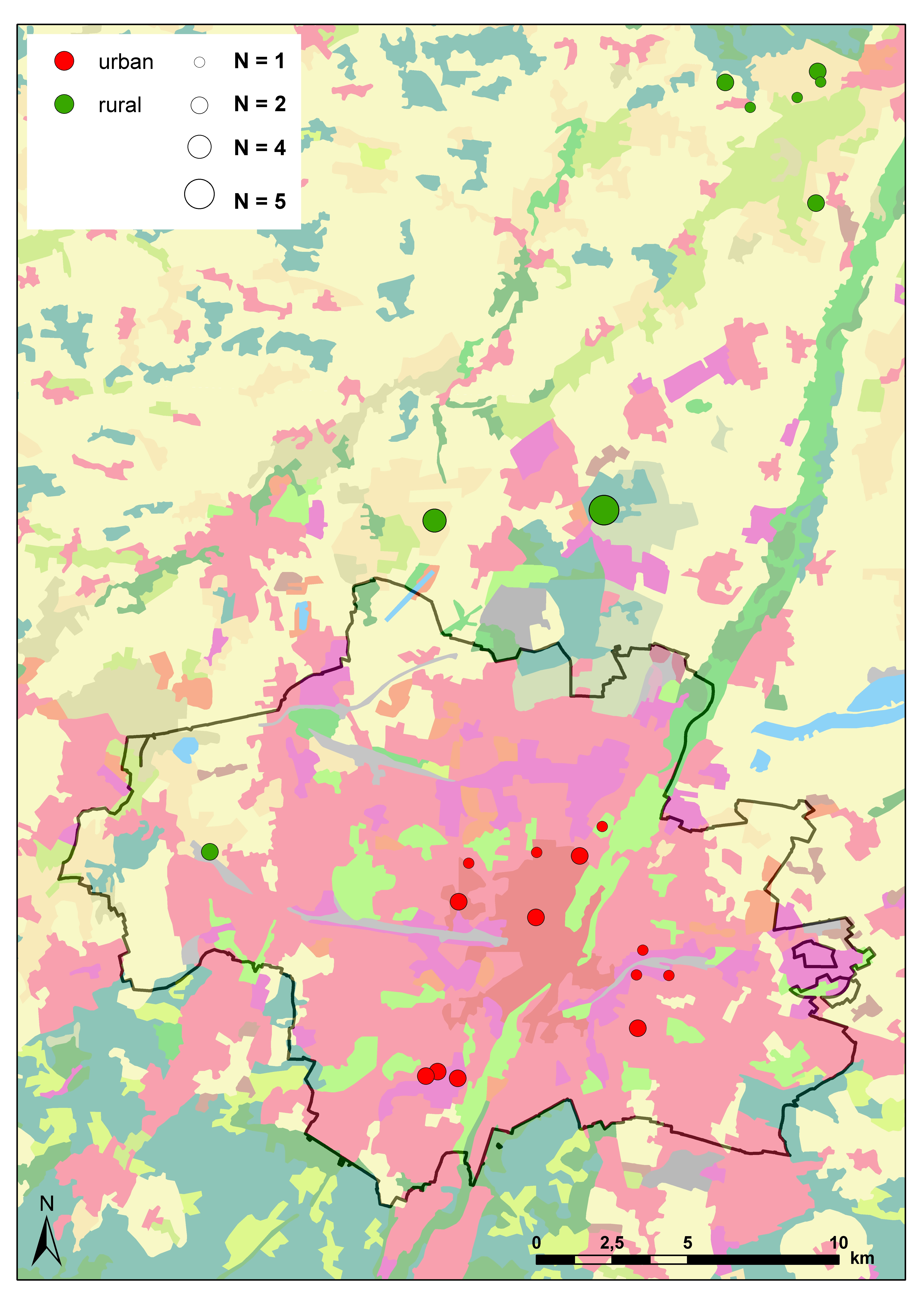

Supplement: Figure S1 — Locations of pollen sampling. Birch pollen were sampled during the birch flowering season of 2010. Red dots represent urban trees, green dots rural trees. Background: CORINE Land Cover 2000 (EEA 2000), major classes: red = urban fabric, green = forest and pastures, yellow = arable land, blue = rivers, lakes (see www.eea.europa.eu/themes/landuse/interactive/clc-download for a complete legend). (TIFF) [file pone.0080147.s001.tiff]

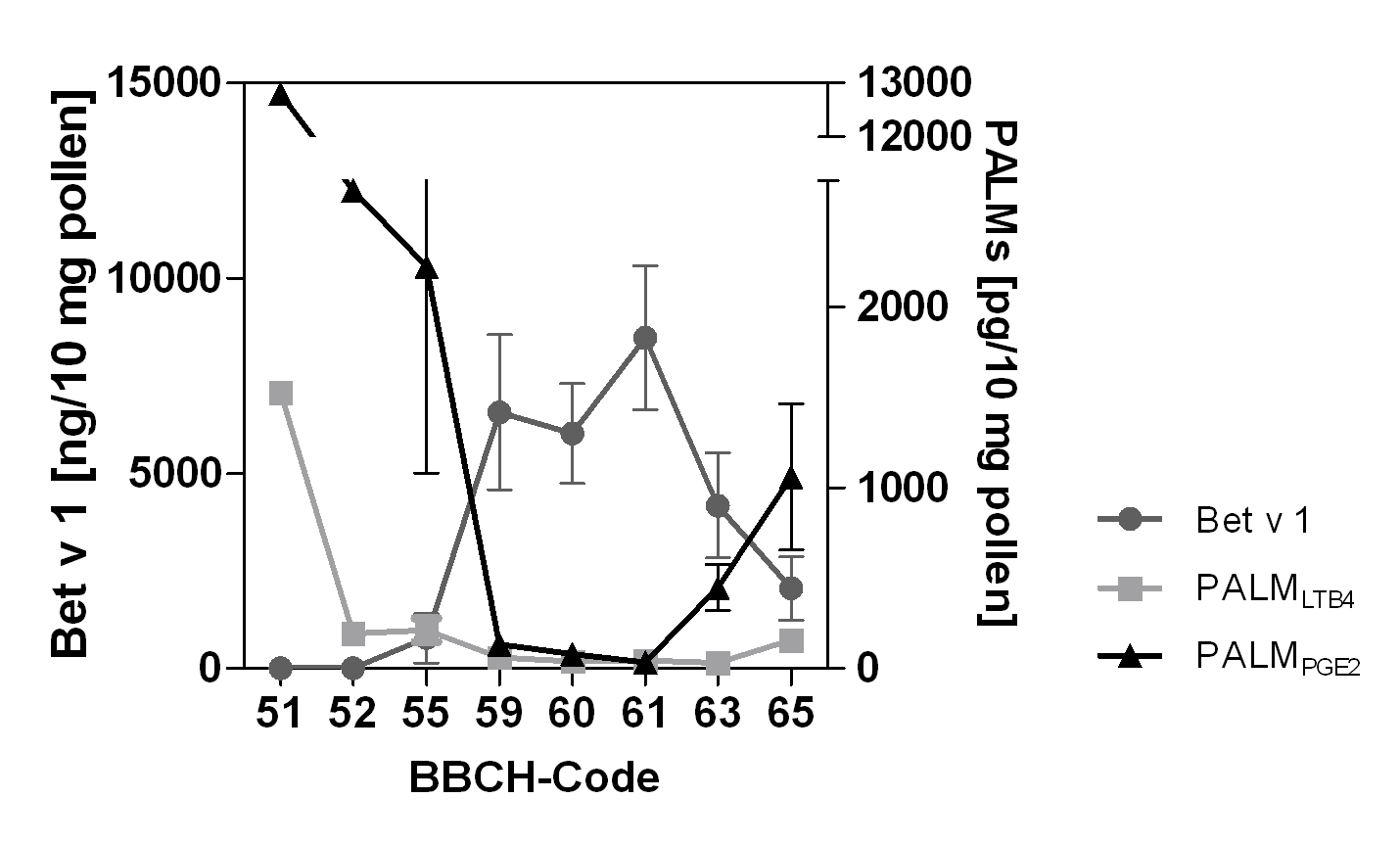

Supplement: Figure S2 — Catkin maturation and allergenic potential of pollen. A: Catkins of different maturation stages were collected at different time points from the same trees (BBCH-Code 51–52: n = 1; BBCH-Code 55–65: n = 5) and classified according to a BBCH-Code. Pollen were isolated from the catkins and aqueous pollen extracts were prepared. APEs were then analyzed for the presence of Bet v 1 and PALMs. The content of Bet v 1 peaked at maturation stages 60–61. Inversely to Bet v 1, levels of PALMLTB4 and PALMPGE2 were high in pollen from immature catkins and decreased during maturation. A concentration minimum of PALMs corresponded to a maximum in Bet v 1. BBCH-Code: 52: catkins increase in length and show green expansion cracks; 55: enhanced expansion cracks through further increase in length; 60: first catkins emit pollen (sporadically); 61: beginning of flowering: few catkins emit pollen; 65: full flowering: more than 50% of the catkins emit pollen; 67: flowering finishing: just a few catkins still emit pollen. (TIFF) [file pone.0080147.s002.tiff]

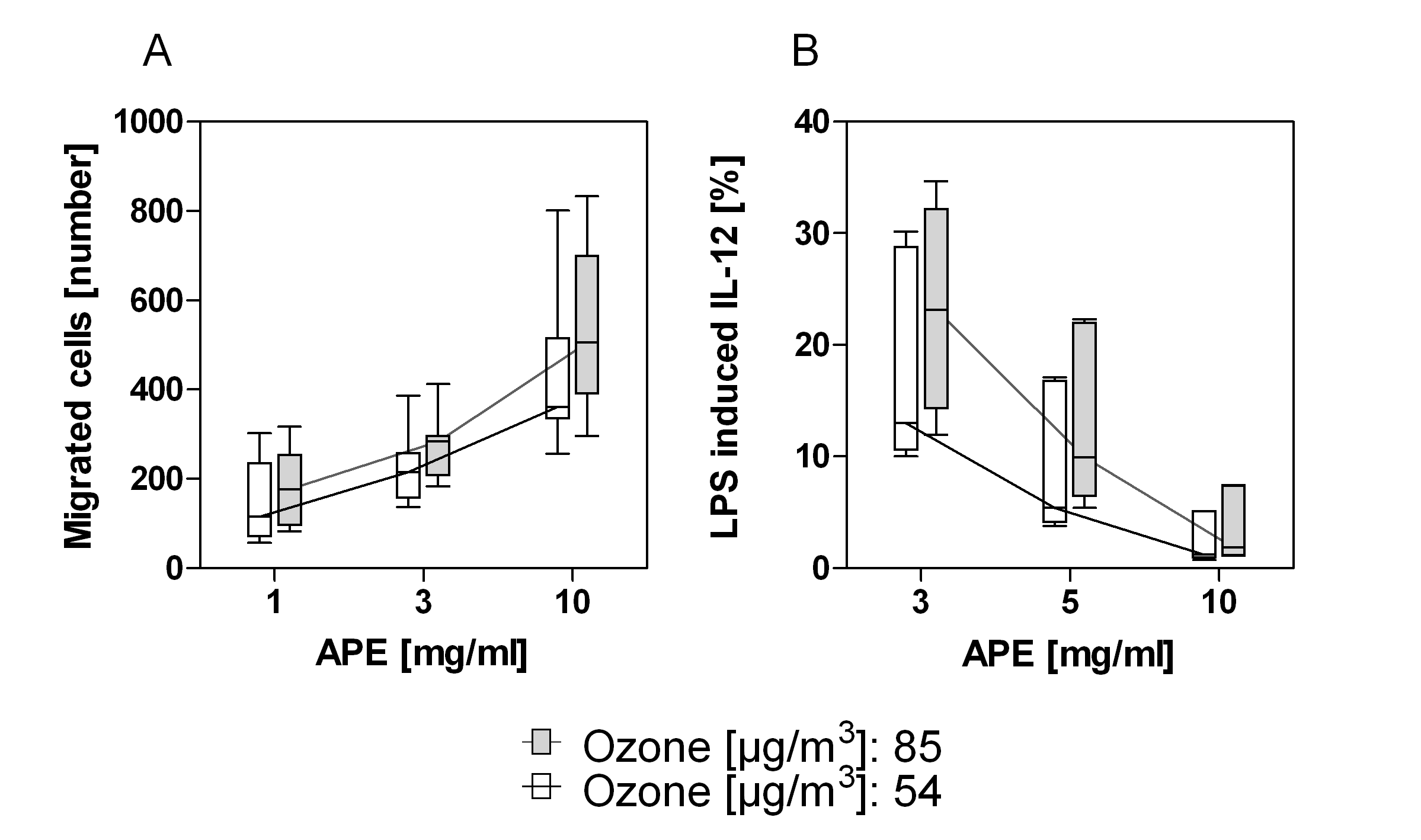

Supplement: Figure S3 — Immune stimulatory versus immune modulatory potential of high versus low ozone-exposed pollen samples. Aqueous extracts (APEs) of birch pollen sampled from high and low ozone exposed trees were chosen for neutrophil migration assays and stimulation of monocyte derived dendritic cells. APEs were applied in 3 concentrations. Higher ozone-exposed pollen induced stronger neutrophil chemotaxis compared to pollen samples from lower ozone–exposed trees (A). In contrast, birch pollen from lower ozone-exposed trees were more potent in inhibiting the LPS-induced release of IL-12p70 from human monocyte-derived dendritic cells (B). APEs were prepared from birch pollen sampled from higher ozone-exposed trees (n = 2; mean ozone: 85 µg/m3) and from lower ozone-exposed trees (n = 2; mean ozone: 54 µg/m3). All APEs were tested in n = 3 patients. *: p<0.05 (Wilcoxon matched-pairs signed-ranks test). (TIFF) [file pone.0080147.s003.tiff]

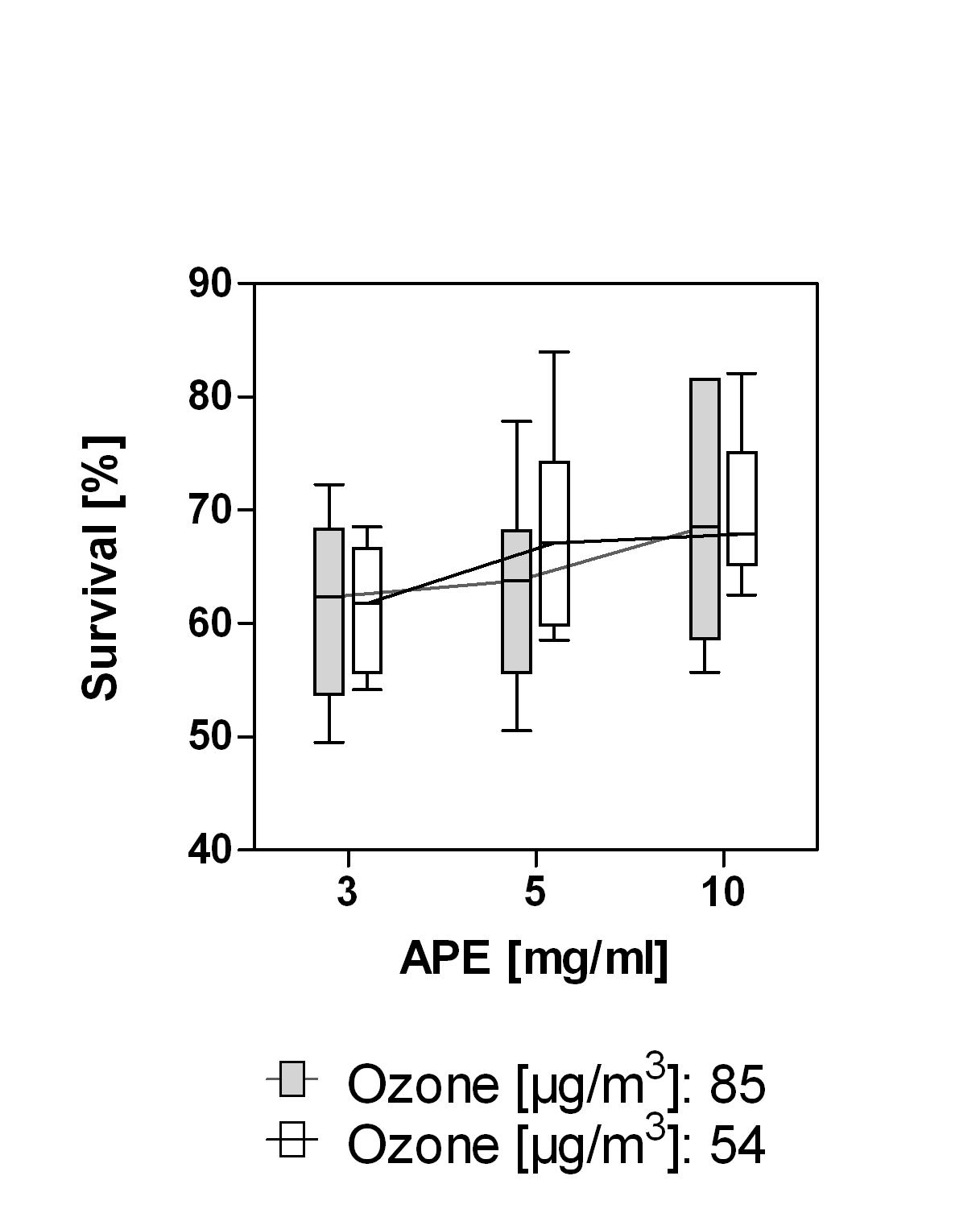

Supplement: Figure S4 — Viability of moDCs after stimulation with LPS plus APEs from high- and low ozone-exposed pollen. Viability of monocyte-derived dendritic cells (moDcs) after 24 h of stimulation with LPS (100 ng/ml) and APEs (1, 3, 10 mg/ml) was tested by propidium iodide staining and subsequent FACS analysis. APEs were prepared from birch pollen sampled from higher ozone-exposed trees (n = 2; mean ozone: 85 µg/m3) and from lower ozone-exposed trees (n = 2; mean ozone: 54 µg/m3). All APEs were tested in n = 3 patients. (TIFF) [file pone.0080147.s004.tiff]
